# Amyloid-Mediated Sequestration of Essential Proteins Contributes to Mutant Huntingtin Toxicity in Yeast

**DOI:** 10.1371/journal.pone.0029832

**Published:** 2012-01-11

**Authors:** Natalia V. Kochneva-Pervukhova, Alexander I. Alexandrov, Michael D. Ter-Avanesyan

**Affiliations:** A. N. Bach Institute of Biochemistry, Russian Academy of Sciences, Moscow, Russia; University of Kent, United Kingdom

## Abstract

**Background:**

Polyglutamine expansion is responsible for several neurodegenerative disorders, among which Huntington disease is the most well-known. Studies in the yeast model demonstrated that both aggregation and toxicity of a huntingtin (htt) protein with an expanded polyglutamine region strictly depend on the presence of the prion form of Rnq1 protein ([*PIN* ^+^]), which has a glutamine/asparagine-rich domain.

**Principal Findings:**

Here, we showed that aggregation and toxicity of mutant htt depended on [*PIN* ^+^] only quantitatively: the presence of [*PIN* ^+^] elevated the toxicity and the levels of htt detergent-insoluble polymers. In cells lacking [*PIN* ^+^], toxicity of mutant htt was due to the polymerization and inactivation of the essential glutamine/asparagine-rich Sup35 protein and related inactivation of another essential protein, Sup45, most probably via its sequestration into Sup35 aggregates. However, inhibition of growth of [*PIN* ^+^] cells depended on Sup35/Sup45 depletion only partially, suggesting that there are other sources of mutant htt toxicity in yeast.

**Conclusions:**

The obtained data suggest that induced polymerization of essential glutamine/asparagine-rich proteins and related sequestration of other proteins which interact with these polymers represent an essential source of htt toxicity.

## Introduction

Expansion of polyglutamine (polyQ) stretches in nine otherwise unrelated human proteins causes neurodegenerative diseases accompanied by deposition of amyloid protein aggregates formed by these proteins. One of the most common polyQ disorders, Huntington disease, is caused by exon I *IT15* mutations that increase the number of CAG triplets, coding for Q in the huntingtin (htt) protein and develops at a probability proportional to the number of these repeats. Htt with expanded N-terminal polyQ can aggregate and form insoluble intracellular inclusions which appear mostly in the nucleus and, to a lesser extent, in the cytoplasm [1]. Despite extensive studies, the molecular bases of polyQ diseases are still unclear, though it was shown that the toxic effect of expanded polyQ proteins is related to interference with the normal function of cellular proteins thus affecting various cellular processes. Indeed, pathological htt impairs gene transcription, ubiquitin-proteasome system, causes mitochondrial dysfunction, dysregulation of Ca^2+^ homeostasis, impairment of axonal transport and genotoxic stress (see [2] for a review).

PolyQ disorders and Huntington disease in particular are especially attractive for modeling in yeast, because, similar to them, yeast prions rely on domains enriched with Q. As in humans, in yeast aggregation and toxicity of htt increase with polyQ length and targeting of mutant htt into the nucleus alters transcription of a subset of genes and decreases cell viability [3]. Besides glutamine, prion domains of yeast proteins are also rich in asparagine residues (N). Importantly, expanded polyN stretches are similar to polyQ in their propensity to form aggregates in yeast and were also used to model polyQ disorders since nuclear localization of such proteins also causes transcriptional abnormalities and cell death [4]. Cytoplasmically expressed htt with an expanded polyQ region was also shown to be toxic, although its toxicity and aggregation depended on the presence of [*PIN* ^+^], the prion form of the QN-rich protein Rnq1, known to facilitate the *de novo* appearance of other yeast prions [5], [6]. Besides, toxicity of expanded htt in yeast cytoplasm is modulated by the sequences flanking the polyQ stretch [7]. Use of the yeast model revealed that polyQ aggregation results in endocytosis impairment, suggesting that this is one of the causes of cellular toxicity [8]. Another reason for toxicity of proteins with expanded polyQ can be interactions with certain genome-encoded Q/N-rich proteins whose lack or overproduction can cause or abolish polyQ toxicity [6].

Previously we have shown that aggregation of proteins with expanded polyQ, including mutant htt, in the cytoplasm of yeast cells caused polymerization of chromosomally-encoded Q/N-rich proteins [9]. Since there are a large number of proteins with long Q/N-rich stretches in both humans and yeast [10], [11], it is likely that at least some of them would efficiently polymerize in response to the accumulation of polyQ amyloids, which may cause depletion of their functional soluble form and, as a result, cell death, if these proteins are essential. Here, we used the yeast model of Huntington disease to confirm this suggestion for the essential Q/N-rich protein, translation termination factor eRF3, which is usually designated in yeast as Sup35.

## Results

### [*PIN* ^+^]-independent polymerization of 103Q-GFP

**Figure 1:**
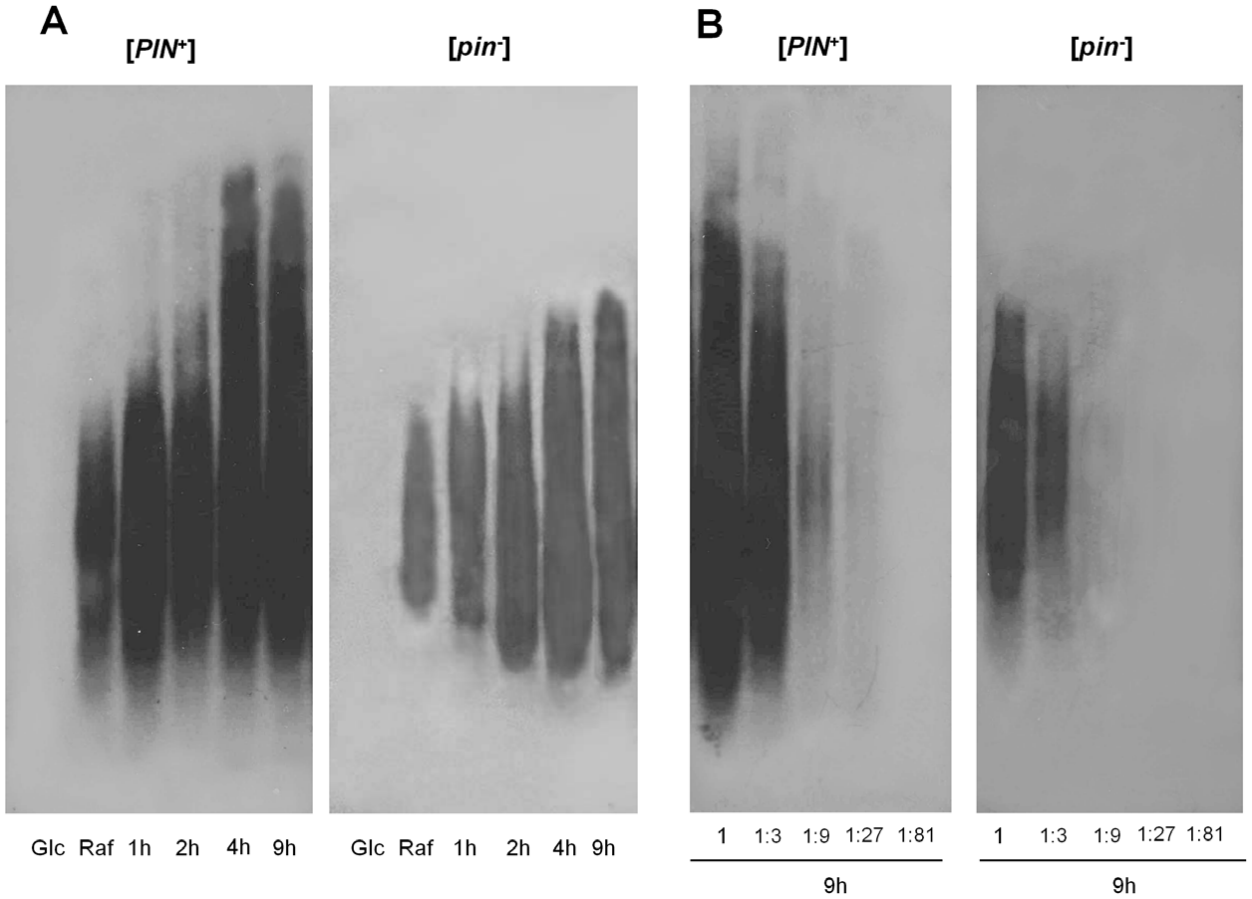
Time-dependent appearance of the 103Q-GFP SDS-insoluble polymers. (**A**) Cells of the [*psi* ^−^] [*PIN* ^+^] or [*psi* ^−^] [*pin* ^−^] transformants of the strain 74-D694 with the multicopy p103Q-GFP plasmid were grown in liquid SC-Ura glucose-containing medium (Glc), then in the same selective raffinose-containing (Raf) and galactose-containing (Gal) media, as described in Materials and Methods, and incubated in the latter medium for 1, 2, 4, 6 and 9 h. (**B**) 1∶3, 1∶9, 1∶27 and 1∶81, dilutions of the sample taken after 9 h incubation. Polymers of 103Q-GFP were visualized by SDD-AGE. Blots were stained with the monoclonal anti-GFP antibody.

In yeast, aggregation and toxicity of overproduced htt with expanded polyQ requires the presence of either [*PIN* ^+^] or [*PSI* ^+^] prions [5], [6]. This was shown for htt with a polyQ stretch consisting of 103 Q (103Q) fused to the green fluorescent protein (GFP), which allows monitoring of 103Q-GFP aggregation microscopically, as distinct fluorescent foci. However, as we have shown previously, polyQ proteins could also form SDS-insoluble polymers in cells lacking these prions ([*psi* ^−^] [*pin* ^−^] cells) [9], [12]. This prompted us to study the dependence of 103Q-GFP polymerization on the [*PIN* ^+^] prion. In agreement with published data, the ability of 103Q-GFP to form fluorescent foci depended on [*PIN* ^+^]: in the absence of this prion 103Q-GFP aggregates were rarely found, while the same protein but with a stretch of 25 Q, 25Q-GFP, did not form aggregates neither in [*PIN* ^+^] nor [*pin* ^−^] (data not shown). However, SDD-AGE analysis showed that overproduced 103Q-GFP formed polymers in [*pin* ^−^] cells, though approximately 3-fold less efficiently than in [*PIN* ^+^] cells (Fig. 1).

**Figure 2:**
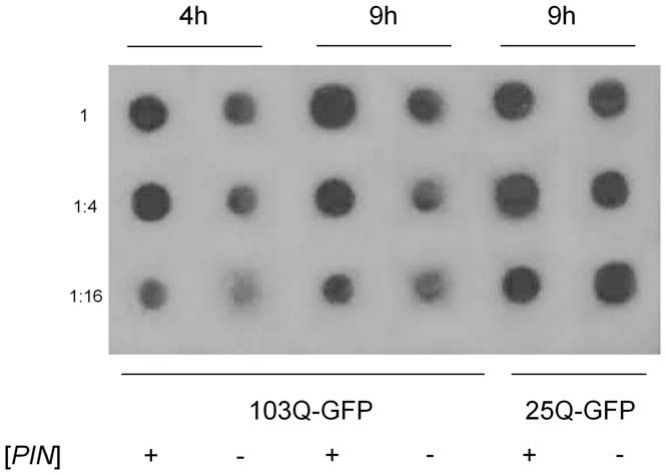
Semi-quantitative dot-blot analysis of 103Q-GFP and 25Q-GFP. Transformants of 74-D694 [*psi* ^−^] [*PIN* ^+^] or [*psi* ^−^] [*pin* ^−^] with the multicopy p103Q-GFP or p25Q-GFP plasmids were grown as described in Materials and Methods with incubation in SC-Ura Gal medium for indicated time. Equal amounts of total protein (confirmed by staining the same membranes by Ponceau S, a non-specific protein stain) from each lysate were serially diluted in four-fold increments and applied to a nitrocellulose membrane. Blots were probed with the monoclonal anti-GFP antibody.

The difference in the 103Q-GFP polymer abundance in [*PIN* ^+^] and [*pin* ^−^] cells could be due to the influence of [*PIN* ^+^] on the levels of this protein (Fig. 2). Most likely this was not due to increased expression of 103Q-GFP, since [*PIN* ^+^] did not affect the levels of 25Q-GFP, which did not form SDS-insoluble polymers in either [*PIN* ^+^] or [*pin* ^−^] cells (data not shown), despite the 25Q-GFP-encoding gene being under the control of the same promoter as that of 103Q-GFP. Therefore, one can suggest that higher levels of 103Q-GFP in [*PIN* ^+^] cells compared with [*pin* ^−^] were due to accelerated accumulation of its polymers (Fig. 1) which are less susceptible to proteolysis than the soluble form of this protein. Interestingly, 25Q-GFP levels in both [*PIN* ^+^] and [*pin* ^−^] cells were higher than those of 103Q-GFP in [*PIN* ^+^], which could reflect a lesser stability of 103Q-GFP.

### Effect of [*PIN* ^+^] on 103Q-GFP-induced polymerization of Rnq1 and Sup35

**Figure 3:**
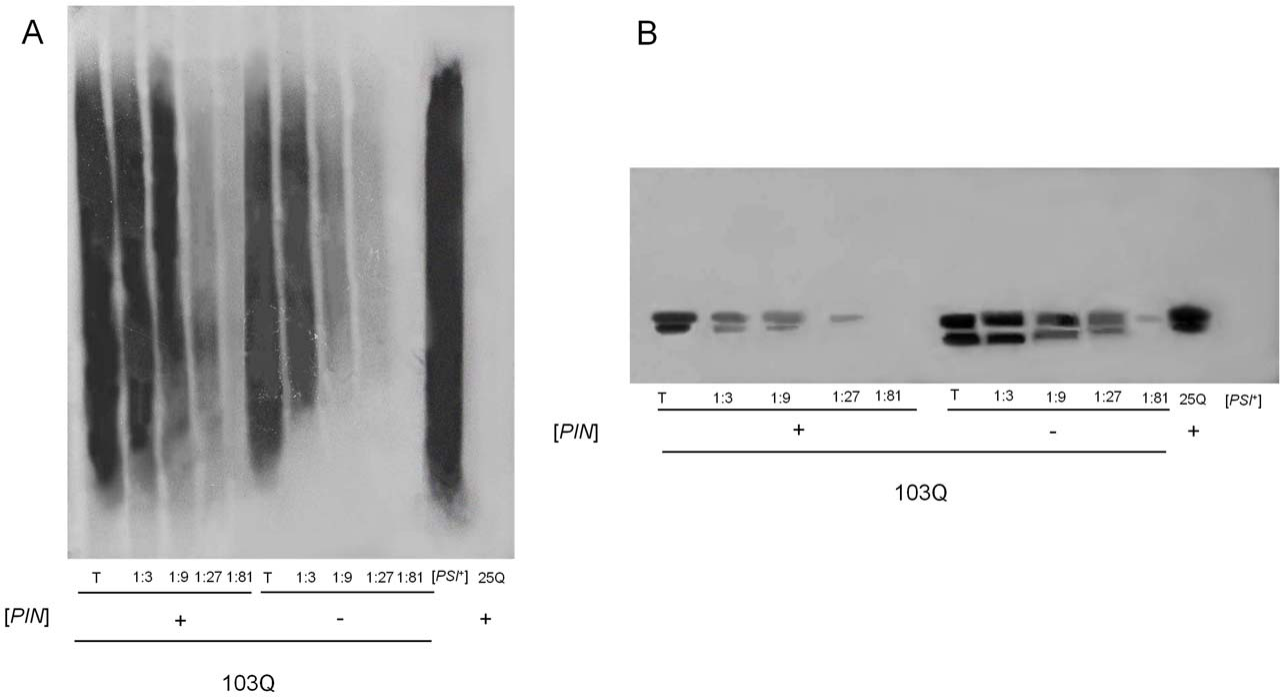
103Q-GFP-dependent polymerization of Sup35. (**A**) Polymers of Sup35 visualized by SDD-AGE. 74-D694 [*psi* ^−^] [*PIN* ^+^] and [*psi* ^−^] [*pin* ^−^] transformants with the p103Q-GFP plasmid were grown as described in Materials and Methods. After incubation in SC-Ura Gal medium for 9 h, cells were harvested and their lysates were used to analyze the amount of SDS-insoluble polymers of Sup35. (**B**) The levels of Sup35 monomer, SDS-PAGE analysis. Lysates were obtained from cells grown as described above. The samples were not boiled before loading onto the gel which only allowed SDS-soluble Sup35 to enter the gel [34]. Lower bands represent a Sup35 degradation product, characteristic of the non-prion/amyloid form of Sup35 [36]. Total lysates (T) and their serial dilutions are indicated. 74-D694 [*psi* ^−^] [*pin* ^−^] expressing 25Q-GFP and 74-D694 [*PSI* ^+^], respectively, are shown for comparison. Blots were stained with anti-Sup35NM antibody. Quantification of Sup35 by densitometric analysis demonstrated that upon expression of 103Q-GFP the levels of soluble Sup35 were approximately 3-fold lower in [*PIN* ^+^] than in [*pin* ^−^] cells.

**Figure 4:**
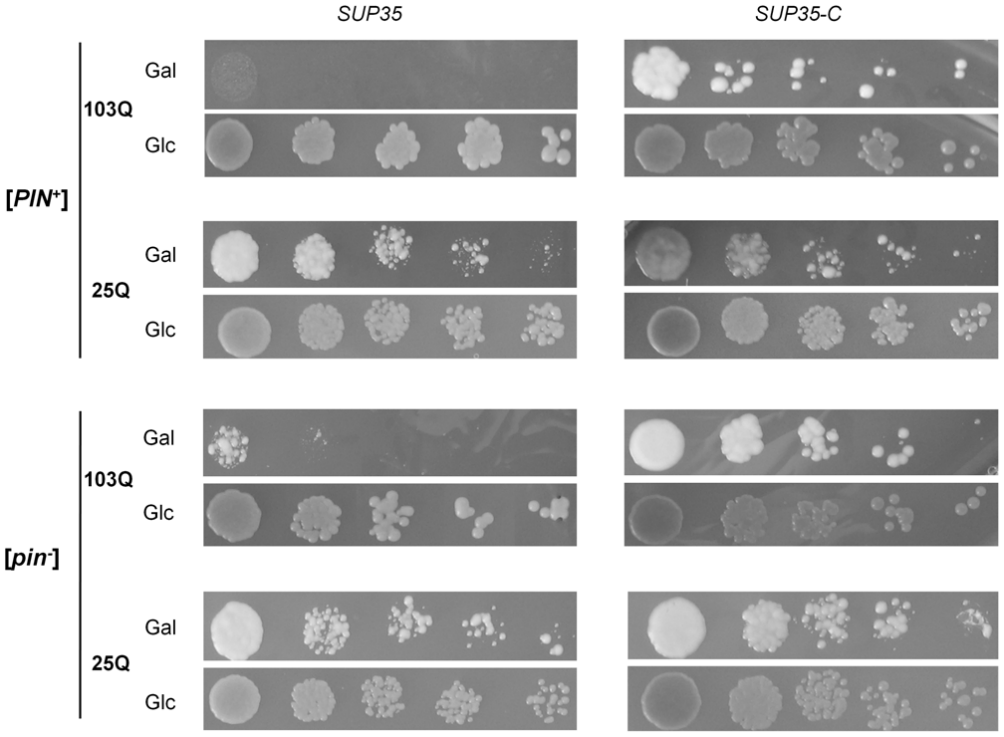
PolyQ toxicity depends on [*PIN* ^+^] and Sup35. The [*psi* ^−^] [*PIN* ^+^] and [*psi* ^−^] [*pin* ^−^] transformants of the 74-D694 strain (*SUP35*) or its 74-D694 ΔS35 derivative disrupted for chromosomal *SUP35* and carrying *SUP35-C* on a centromeric plasmid (*SUP35-C*), both expressing either 103Q-GFP or 25Q-GFP, were grown at 30°C in liquid SC-Ura medium with glucose resuspended in the same medium but with raffinose instead of glucose and after 12 h incubation, cell suspensions were diluted to an OD_600_ of 1.0, and then spotted onto SC-Ura plates with galactose as a sole carbon source (Gal) and incubated for 4 days. Equal spotting was controlled by spotting the cells onto SC-Ura plates containing glucose as carbon source (Glc) in parallel. Five serial 5-fold dilutions of cell suspensions are shown.

Previously we have demonstrated that SDS-insoluble polymers of 103Q-GFP can seed polymerization of Sup35 in [*PIN* ^+^] cells [9]. Here we showed that polymers of 103Q-GFP also seeded Sup35 (Fig. 3A) and Rnq1 polymerization (data not shown) in [*pin* ^−^] cells. The amount of Sup35 polymers correlated with the levels of 103Q-GFP polymers and was approximately 3-fold higher in [*PIN* ^+^] as compared to [*pin* ^−^] cells. Correspondingly, the amount of soluble Sup35 was inversely proportional to the levels of its polymerized form being approximately 3-fold higher in [*pin* ^−^] than in [*PIN* ^+^] cells (Fig. 3B), which caused a detectable increase of nonsense codon readthrough (Fig S1). In contrast to 103Q-GFP, overproduction of non-polymerizing 25Q-GFP neither inhibited growth of [*PIN* ^+^] and [*pin* ^−^] strains (Fig. 4) nor caused appearance of Sup35 polymers in [*PIN* ^+^] and [*pin* ^−^] cells (Fig. 3A and data not shown).

### 103Q-GFP toxicity partially depends on depletion of Sup35 and Sup45

Unlike Rnq1, Sup35 is an essential protein and therefore depletion of its functional, soluble form should be deleterious for the cell. In agreement with this, though overproduction of 103Q-GFP caused growth inhibition of both [*PIN* ^+^] and [*pin* ^−^] strains, this effect was more pronounced in a [*PIN* ^+^] background (Fig. 4) in which both 103Q-GFP and Sup35 polymerized most efficiently (Fig. 1 and 3). Expression of the non-aggregating form of Sup35, which lacked the NM region (Sup35C), instead of the full-length protein, alleviated the toxic effect of 103Q-GFP in [*PIN* ^+^] cells and completely abolished it in [*pin* ^−^] cells (Fig. 4). This suggested that Q103-GFP toxicity was due to depletion of soluble Sup35. Alternatively, it was possible that this effect was due to the influence of Sup35C on the levels of 103Q-GFP and Rnq1 polymers or due to effects of Sup35C on cell growth irrespective of the presence of these polymers. However since neither the levels nor size distributions of Rnq1 and 103Q-GFP polymers did not depend on the presence of the NM region in Sup35 (Fig. S2), and Sup35C did not affect growth of cells not expressing 103Q-GFP (Fig. S3), these possibilities seemed unlikely. Importantly, there were also other causes for toxicity of 103Q-GFP in [*PIN* ^+^] cells, since a [*PIN* ^+^] strain expressing the non-polymerizing Sup35C protein still manifested a slight growth defect.

**Figure 5:**
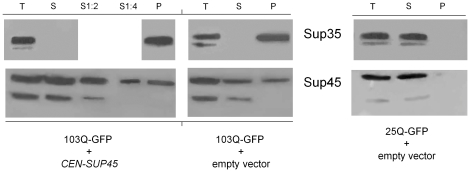
Centrifugation analysis of aggregation of Sup35 and Sup45 in the presence of 103Q-GFP or 25Q-GFP. Transformants of the strain 74-D694 [*psi* ^−^] [*PIN* ^+^] with plasmids expressing 103Q-GFP or 25Q-GFP were grown as described in Materials and Methods. After incubation in SC-Ura Gal medium for 9 h, cells were harvested. Cell lysates were fractionated by centrifugation and fractions were analyzed by Western blotting: staining with either anti-Sup35NM (Sup35) or anti-Sup45 (Sup45) polyclonal antibodies. The lower bands in the Sup35 image represent a degradation product (see legend to Fig. 3); the lower bands in the Sup45 image are nonspecific protein staining [20]. T, total protein; S, soluble fraction (1∶2 and 1∶4, dilutions); P, pellet. CEN-SUP45, the centromeric plasmid pRS315-SUP45; empty vector, pRS315. Quantitative data on Sup45 levels obtained by densitometric analysis demonstrated that expression of Q103-GFP caused an approximately 2-fold depletion of the soluble fraction of Sup45, with the remaining portion of the protein being relocated into the pellet fraction. Presence of pRS315-SUP45 caused an approximately 4-fold increase in the level of soluble Sup45 in Q103-GFP expressing cells as compared with the same cells with an empty vector.

**Figure 6:**
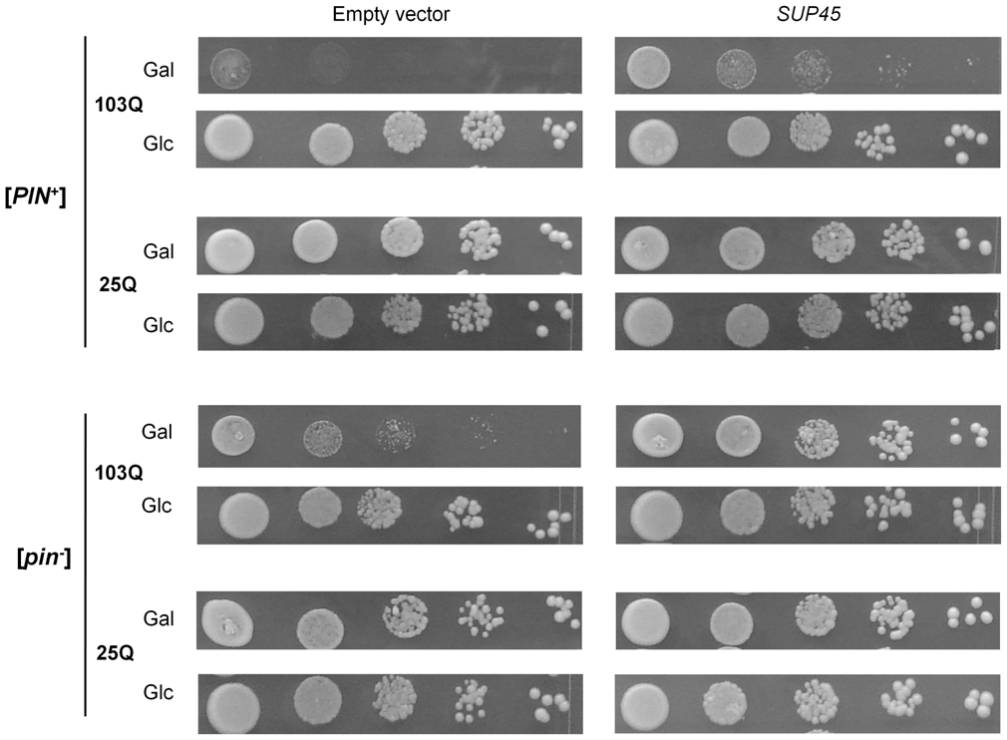
PolyQ toxicity is modulated by the levels of Sup45. Growth of the [*psi* ^−^] [*PIN* ^+^] and [*psi* ^−^] [*pin* ^−^] transformants of the 74-D694 strain carrying either a centromeric plasmid with *SUP45* or an empty vector as a control and expressing either 103Q-GFP or 25Q-GFP, was analyzed as described in the legend to Fig. 4.

It is known that Sup35 prion aggregates may include different proteins able to interact with Sup35 [13], [14] in an SDS-sensitive manner. Sequestration of Sup45 (translation termination factor eRF1) into Sup35 prion aggregates can cause growth inhibition, and this defect can be alleviated by increasing Sup45 levels [15], [16]. Similarly to prion polymers of Sup35, non-prion polymers of this protein also contained Sup45, since centrifugation of lysates of [*PIN* ^+^] cells overproducing 103Q-GFP revealed appearance of Sup45 in the pellet fraction and a related 2-fold decrease of the levels of its soluble form, while in cells expressing non-aggregating 25Q-GFP Sup45 was primarily soluble (Fig. 5). Introduction of a centromeric plasmid with *SUP45* into [*PIN* ^+^] and [*pin* ^−^] cells expressing 103Q-GFP partially alleviated the toxic effects of 103Q-GFP (Fig. 6), which could be related to increased levels of soluble Sup45 (Fig. 5).

## Discussion

Earlier it was reported that in contrast to [*PIN* ^+^] cells, cells lacking this prion rarely possess fluorescently detectable aggregates of overproduced 103Q-GFP and exhibit no decreased viability, while protein with a shorter polyQ stretch, 25Q-GFP, does not form aggregates and manifest toxicity in ether [*PIN* ^+^] or [*pin* ^−^] cells [7], [8]. This indicated that the aggregation and toxicity of htt in yeast depends both on the polyQ expansion and the presence of the [*PIN* ^+^] prion. We also observed that 103Q-GFP in [*pin* ^−^] cells and 25Q-GFP in both [*pin* ^−^] and [*PIN* ^+^] cells formed bright fluorescent foci only occasionally, while 103Q-GFP formed numerous visible aggregates in a large proportion of [*PIN* ^+^] cells. However, SDD-AGE polymer analysis led us to another conclusion. While this analysis confirmed the inability of overproduced 25Q-GFP to polymerize in both [*pin* ^−^] and [*PIN* ^+^] cells, 103Q-GFP was shown to form polymers irrespective of the [*PIN* ^+^] state, albeit more polymers were observed in [*PIN* ^+^] than in [*pin* ^−^] cells. In agreement with this, the GFP^N104^ protein (N-terminal fusion of GFP to the sequence of 104 N) formed SDS-insoluble aggregates in the cytoplasm of [*pin* ^−^] cells, which could not be visualized microscopically [4]. Thus, despite the convenience of the GFP test for aggregate detection, to avoid misinterpretations, it should be supported by alternative approaches.

Contrary to earlier studies, we observed that overproduced 103Q-GFP inhibited growth of both [*PIN* ^+^] and [*pin* ^−^] cells, though this effect was more pronounced in [*PIN* ^+^] cells, in which 103Q-GFP polymerized most efficiently. Importantly, the toxic effect of overproduced 103Q-GFP was related to Sup35 polymerization, since expression of a non-aggregating Sup35C variant of this protein abolished 103Q-GFP toxicity in [*pin* ^−^] cells and reduced it in isogenic [*PIN* ^+^] cells. Finally, the toxicity of 103Q-GFP also depended on sequestration of Sup45 into Sup35 aggregates. The critical role of Sup35 in 103Q-GFP toxicity may seem surprising, since it is just one of more than a hundred of yeast Q/N-rich proteins, many of which are essential [10], [11]. However, it is necessary to stress that among essential Q/N-rich proteins, Sup35 has one of the highest expression levels [17] and that its polymerization caused aggregation and inactivation of another essential protein, Sup45. It is also necessary to take into account that 103Q-GFP may seed polymerization of different cellular Q/N-rich proteins with varying efficiency and Sup35 may be among those whose polymerization is seeded most efficiently. At last, proteins enriched in Q and N may differ from each other by their intrinsic propensity to polymerize and Sup35 may be among those which are most prone to polymerization. The contribution of Sup45 depletion to 103Q-GFP toxicity is not surprising, because sequestration of this protein into [*PSI* ^+^] aggregates, but not of other essential proteins able to interact with Sup35, was responsible for growth inhibition of [*PSI* ^+^] cells overproducing Sup35 [14]. Notably sequestration of the translation termination factors could not completely account for 103Q-GFP toxicity, because overproduction of the 103Q-GFP protein also manifested toxicity in the [*PIN* ^+^] strain expressing Sup35C which is unable to form SDS-insoluble polymers.

At present it is believed that microscopically visible protein aggregates, which are frequently associated with neurodegenerative diseases, in fact play a cytoprotective role, while toxicity of amyloidogenic proteins is related to accumulation of their oligomeric species [18], [19]. In this work we observed that in [*pin* ^−^] cells, lacking microscopically visible 103Q-GFP aggregates, toxicity was related to SDS-insoluble aggregates of this protein, which may correspond to toxic oligomers in mammals. At the same time, overproduced 103Q-GFP was more toxic in [*PIN* ^+^] than [*pin* ^−^] cells, which correlated with increased formation of its fluorescently detectable aggregates. However, [*PIN* ^+^] cells also contain more SDS-insoluble polymers not detectable microscopically on their own, which did not allow us to make conclusions about the role of microscopically detectable aggregates of this protein in toxicity.

Notably, depletion of Sup35 and Sup45 induced by 103Q-GFP may impair not only translation termination, but also other processes, since both termination factors have essential non-translational functions, being important for cytoskeleton organization, cytokinesis, cell cycle regulation [20], [21] and probably for other processes [22], [23], such as coupling termination and initiation steps of translation [24], [25] and regulation of mRNA deadenylation and decay [26], which are mediated by interaction of the N-terminal region of Sup35 with poly(A)^+^ binding protein PABP. Modeling of htt toxicity in yeast showed that polymerization of expanded polyQ caused polymerization of cellular Q/N-rich proteins and related sequestration of other proteins able to interact with these Q/N-rich proteins. If such proteins are essential, depletion of their soluble and functional form may be deleterious to the cell. Thus, though there may be different sources of polyQ toxicity in eukaryotes, this mechanism can explain why a wide variety of different processes, including translation, were found to be altered in the yeast model of Huntington disease [27]. It is also likely that a similar mechanism underlies depletion of transcription factors possessing polyQ repeats in mammalian cells expressing proteins with expanded polyQ [28]–[30].

## Materials and Methods

### Plasmids, strains and growth conditions

The strain 74-D694 [*psi* ^−^] [*pin* ^−^] and its [*psi* ^−^] [*PIN* ^+^] derivative [31], as well as 74-D694 ΔS35 [*psi* ^−^] [*PIN* ^+^] and [*psi* ^−^] [*pin* ^−^] with *SUP35* disrupted by the insertion of *TRP1* [12] were used. The plasmids used in this study are described in Table 1. Yeast were grown at 30°C in rich (YPD, 1% yeast extract, 2% peptone, 2% glucose) or synthetic (SC, 0.67% yeast nitrogen base, 2% glucose supplemented with the required amino acids) media. To induce the synthesis of 103Q-GFP and 25Q-GFP chimeric proteins, cell cultures with corresponding plasmids were transferred to liquid selective media with 2% raffinose as a sole carbon source and grown until mid-log phase. Then cultures were transferred to of the same medium with galactose instead of raffinose and cells were grown for the indicated time. The final concentration of galactose in the medium was 2%.

**Table 1:** Plasmids used in this study.

Plasmid	Characteristics	Reference
pRS315	Centromeric *LEU2* vector	[37]
pRS315-SUP45	Same as pRS315, but with *SUP45*	[21]
pRS315-SUP35C	Same as pRS315, but with *SUP35-C*	[22]
p103Q-GFP	Multicopy *URA3* pYES2 plasmid, encoding fusion of 103Q with GFP under the control of *GAL1* promoter	[5]
p25Q-GFP	Multicopy *URA3* pYES2 plasmid, encoding fusion of 25Q with GFP under the control of *GAL1* promoter	[5]

### Preparation of yeast cell lysates

Yeast cultures grown in liquid selective media were harvested, washed in water and lyzed by beating with glass beads (Bullet Blender, Next Advance) in buffer A: 30 mM Tris-HCl, pH 7.4, 150 mM NaCl, 1 mM dithiothreitol and 1% Triton X-100. To prevent proteolytic degradation, 10 mM phenylmethylsulfonyl fluoride and Complete™ protease inhibitor cocktail (Roche Applied Science) were added. Cell debris was removed by centrifugation at 1500 g for 4 min.

### Centrifugation

To separate Sup35 and Sup45 polymer and monomer fractions, 500 µl of yeast cell lysates were centrifuged at 100,000 g (48,000 rpm in a Ti75 rotor, Beckman Optima TL ultracentrifuge) for 1 h at 4°C.

### Electrophoresis and blotting

These were performed as described previously [32]–[34]. Protein loads were equalized for each gel. For analysis of amyloid polymers we used horizontal 1.8% agarose gels in the Tris-Acetate-EDTA (TAE) buffer with 0.1% SDS. Lysates were incubated in the sample buffer (0.5× TAE, 2% SDS, 5% glycerol and 0.05% Bromophenol Blue) for 5 min at 37°C. After the electrophoresis, proteins were transferred from gels to nitrocellulose membrane sheets (ThermoScientific, USA) by vacuum-assisted capillary blotting for 5 h (agarose gels), or electrophoretically (polyacrylamide gels). Bound antibody was detected using the ECL West Dura system (Thermo Scientific). It should be noted that detergents (SDS or sarcosyl) in non-boiled samples increase degradation of Sup35 monomers. This can result in the absence of Sup35 monomer bands in SDD-AGE gels. Rabbit polyclonal antibodies against Sup35 and Sup45 were used. Anti-GFP monoclonal antibody was obtained from Rusbiolink (Russia). Estimation of relative amount of 103Q-GFP or 25Q-GFP in lysates was performed as described in [35], with minor modifications. Densitometry measurements were performed using ImageJ software.

## Supporting Information

Figure S1 **Polymerization of 103Q-GFP causes an increase in nonsense codon readthrough.** The strain 74-D694 [*psi* ^−^][*PIN* ^+^] carrying the *URA3* p25Q-GFP (25Q) or p103Q-GFP (103Q) plasmids was transformed with either the *LEU2* plasmid pUKC815-L (encodes a *PGK1-lacZ* gene fusion) or pUKC817-L (encodes the same gene fusion but with in frame UAA at the junction of the *PGK1* and *lacZ* genes) (Stansfield I, Jones KM, Kushnirov VV, Dagkesamanskaya AR, Poznyakovski AI, Paushkin SV, Nierras CP, Cox BS, Ter-Avanesyan MD, Tuite MF (1995) The products of the *SUP45* (eRF1) and *SUP35* genes interact to mediate translation termination in *Saccharomyces cerevisiae*. EMBO J 14: 4365–4373). Transformants were grown consecutively in liquid glucose-, raffinose- and galactose-containing media selective for the plasmids, and after a 9 h incubation in SC-Ura -Leu Gal medium appropriate aliquots of yeast culture were taken and β-galactosidase activity was assayed. All data represent an average of at least three independent experiments. The nonsense readthrough levels were determined as ratio of β-galactosidase activities in the cells transformed with the plasmid pUKC817-L to that of the transformant with pUKC815-L.
(TIF)

Figure S2 **The levels of Rnq1 and 103Q-GFP polymers do not depend on the presence of the Sup35 NM region.** The 74-D694 [*psi* ^−^] [*PIN* ^+^] strain (*SUP35*) or its 74-D694 ΔS35 derivative disrupted for the chromosomal *SUP35* gene and carrying *SUP35-C* on a centromeric plasmid (*SUP35-C*), both expressing 103Q-GFP, were grown as described in Materials and Methods. After incubation in SC-Ura Gal medium for 9 h cells were harvested and their lysates were used to estimate the amount of Q103-GFP and Rnq1 SDS-insoluble polymers by SDD-AGE analysis. Blots were stained with anti-Rnq1 polyclonal antibody (A) or anti-GFP monoclonal antibody (B).
(TIF)

Figure S3 **Sup35C does not affect growth of cells not expressing 103Q-GFP.** The [*psi* ^−^] [*PIN* ^+^] and [*psi* ^−^] [*pin* ^−^] transformants of the 74-D694 strain (*SUP35*) or its 74-D694 ΔS35 derivative with disruption of chromosomal *SUP35* carrying the centromeric pRS315-SUP35C plasmid (*SUP35-C*), were grown as described in the legend to Fig. 4. Cell suspensions were diluted to an OD_600_ of 1.0, spotted onto Gal and Glc plates and incubated for 4 days. Five serial 5-fold dilutions of cell suspensions are shown.
(TIF)
