# Supplementary figures and images for: Amyloid-Mediated Sequestration of Essential Proteins Contributes to Mutant Huntingtin Toxicity in Yeast
Source: PLoS One. 2012 Jan 11;7(1):e29832. doi: 10.1371/journal.pone.0029832 (PMC3256205; doi:10.1371/journal.pone.0029832)

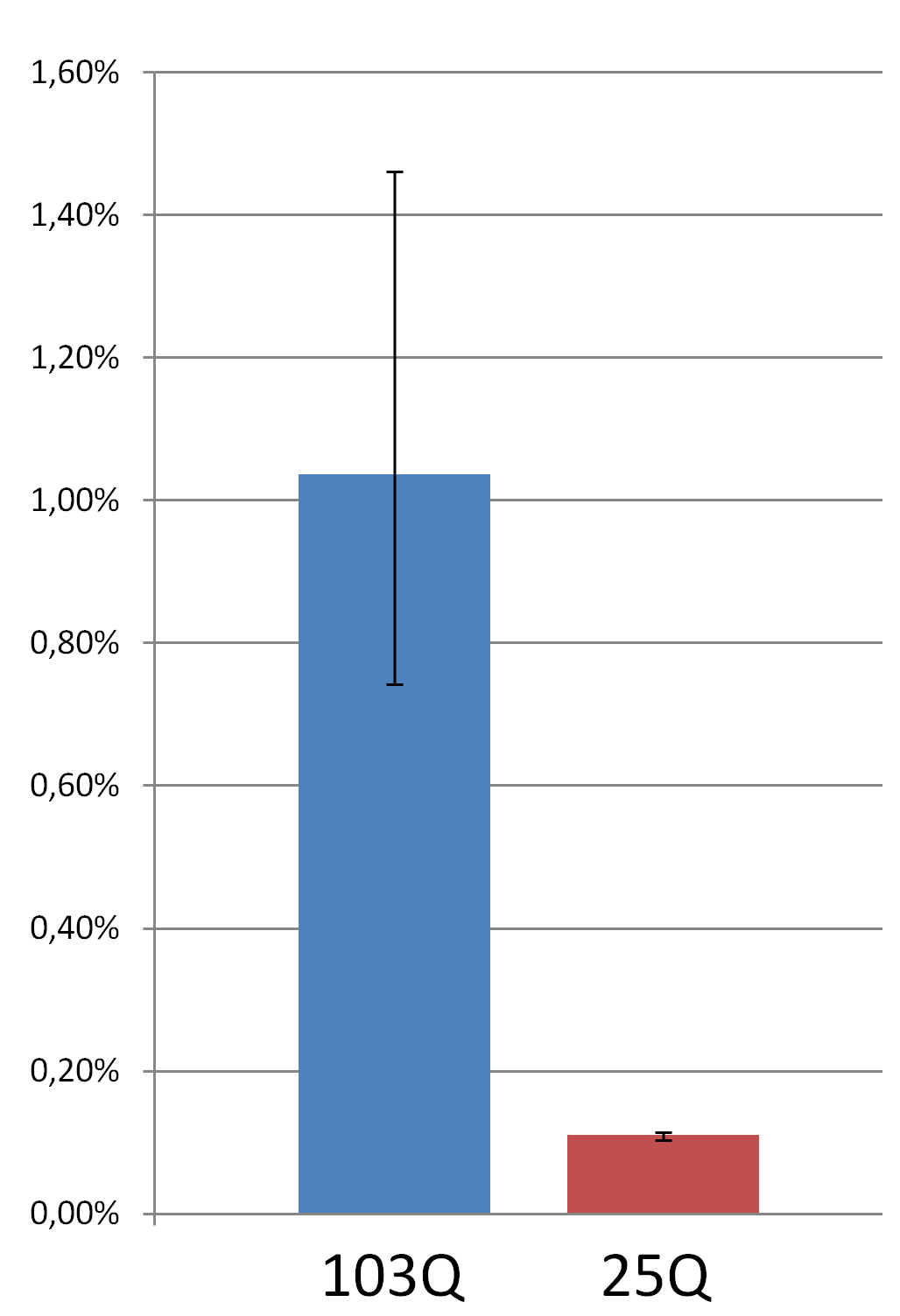

Supplement: Figure S1 — Polymerization of 103Q-GFP causes an increase in nonsense codon readthrough. The strain 74-D694 [psi −][PIN +] carrying the URA3 p25Q-GFP (25Q) or p103Q-GFP (103Q) plasmids was transformed with either the LEU2 plasmid pUKC815-L (encodes a PGK1-lacZ gene fusion) or pUKC817-L (encodes the same gene fusion but with in frame UAA at the junction of the PGK1 and lacZ genes) (Stansfield I, Jones KM, Kushnirov VV, Dagkesamanskaya AR, Poznyakovski AI, Paushkin SV, Nierras CP, Cox BS, Ter-Avanesyan MD, Tuite MF (1995) The products of the SUP45 (eRF1) and SUP35 genes interact to mediate translation termination in Saccharomyces cerevisiae. EMBO J 14: 4365–4373). Transformants were grown consecutively in liquid glucose-, raffinose- and galactose-containing media selective for the plasmids, and after a 9 h incubation in SC-Ura -Leu Gal medium appropriate aliquots of yeast culture were taken and β-galactosidase activity was assayed. All data represent an average of at least three independent experiments. The nonsense readthrough levels were determined as ratio of β-galactosidase activities in the cells transformed with the plasmid pUKC817-L to that of the transformant with pUKC815-L. (TIF) [file pone.0029832.s001.tif]

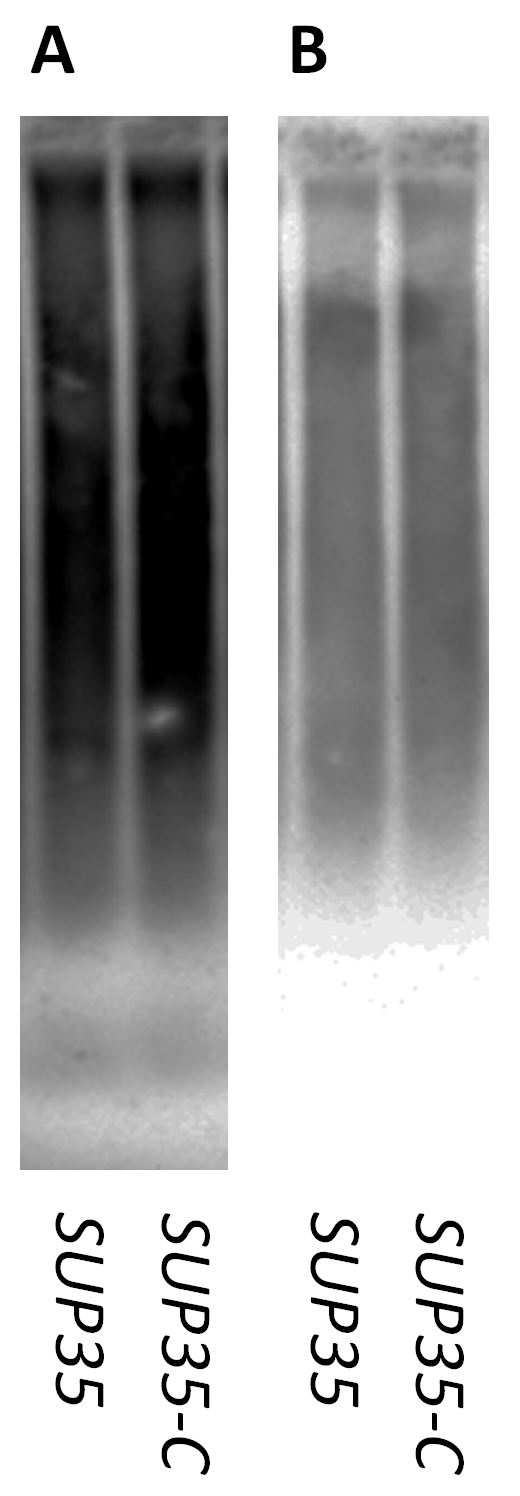

Supplement: Figure S2 — The levels of Rnq1 and 103Q-GFP polymers do not depend on the presence of the Sup35 NM region. The 74-D694 [psi −] [PIN +] strain (SUP35) or its 74-D694 ΔS35 derivative disrupted for the chromosomal SUP35 gene and carrying SUP35-C on a centromeric plasmid (SUP35-C), both expressing 103Q-GFP, were grown as described in Materials and Methods. After incubation in SC-Ura Gal medium for 9 h cells were harvested and their lysates were used to estimate the amount of Q103-GFP and Rnq1 SDS-insoluble polymers by SDD-AGE analysis. Blots were stained with anti-Rnq1 polyclonal antibody (A) or anti-GFP monoclonal antibody (B). (TIF) [file pone.0029832.s002.tif]

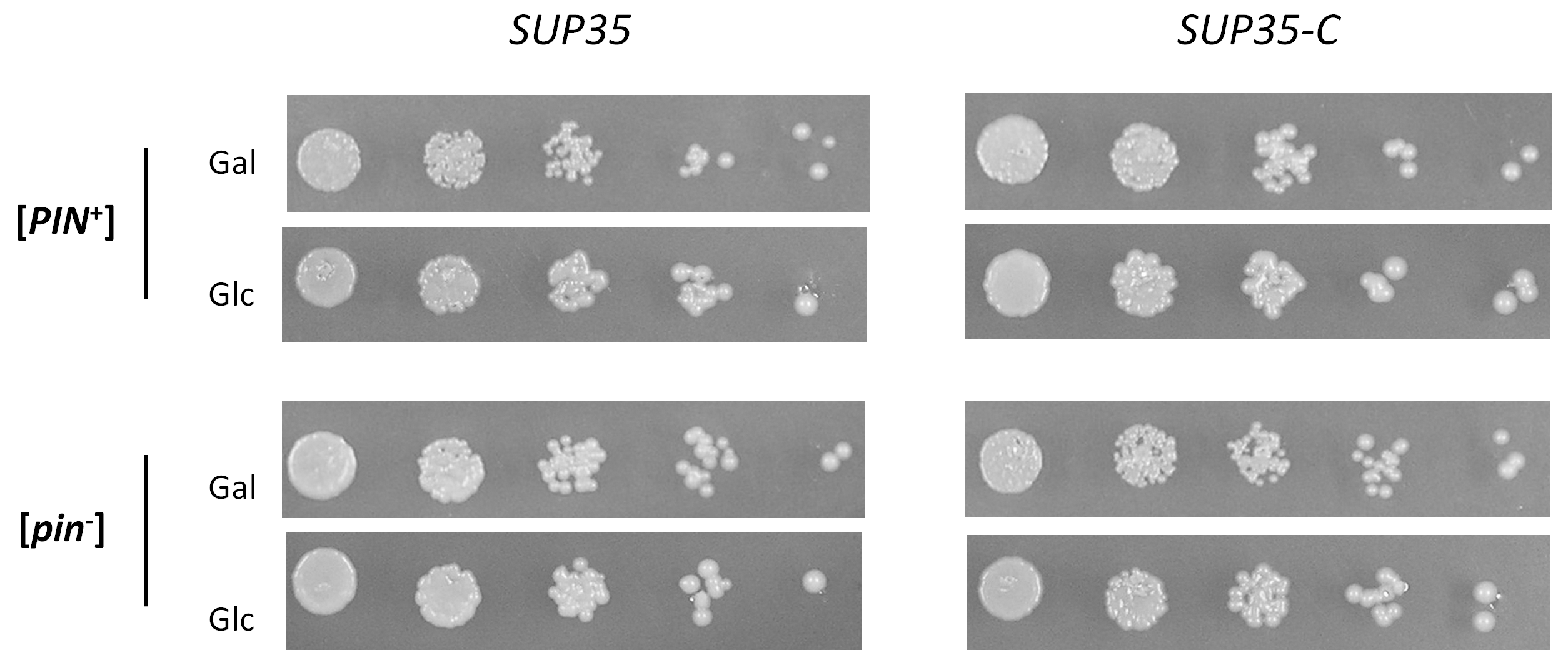

Supplement: Figure S3 — Sup35C does not affect growth of cells not expressing 103Q-GFP. The [psi −] [PIN +] and [psi −] [pin −] transformants of the 74-D694 strain (SUP35) or its 74-D694 ΔS35 derivative with disruption of chromosomal SUP35 carrying the centromeric pRS315-SUP35C plasmid (SUP35-C), were grown as described in the legend to Fig. 4. Cell suspensions were diluted to an OD600 of 1.0, spotted onto Gal and Glc plates and incubated for 4 days. Five serial 5-fold dilutions of cell suspensions are shown. (TIF) [file pone.0029832.s003.tif]
